# Correlation of High-Molecular-Weight Adiponectin and Leptin Concentrations with Anthropometric Parameters and Insulin Sensitivity in Newborns

**DOI:** 10.1155/2014/435376

**Published:** 2014-10-12

**Authors:** Jia Zheng, Xinhua Xiao, Qian Zhang, Lili Mao, Ming Li, Miao Yu, Jianping Xu, Ying Wang

**Affiliations:** Department of Endocrinology, Key Laboratory of Endocrinology, Ministry of Health, Peking Union Medical College Hospital, Diabetes Research Center of Chinese Academy of Medical Sciences and Peking Union Medical College, No. 1 Shuaifuyuan, Wangfujing Street, Dongcheng District, Beijing 100730, China

## Abstract

*Objective*. High-molecular-weight adiponectin (HMW-adiponectin) and leptin are two important adipokines. The aim of this study was to examine the association between the two adipokines and anthropometric measurements of neonates at birth. Furthermore, we would like to explore whether HMW-adiponectin and leptin correlate with insulin sensitivity in neonates.* Methods*. Venous cord blood samples were obtained from 266 full-term healthy neonates consecutively born at Peking Union Medical College Hospital. HMW-adiponectin, leptin, blood glucose, and insulin concentrations were measured.* Results*. HMW-adiponectin and leptin were significantly higher in females compared with males (*P* = 0.031 and *P* = 0.000, resp.). Univariate correlation analysis showed that leptin concentrations in cord blood were positively associated with gestational age, birth weight, body length, ponderal index, placenta weight, insulin, and insulin sensitivity (all *P* < 0.001). However, there was no correlation between cord blood HMW-adiponectin levels and foetal anthropometric measurements or foetal insulin sensitivity indicators (all *P* > 0.05). Multivariate linear regression analysis indicated that leptin (*B* = −0.126, *P* = 0.045) in cord blood was independently associated with insulin sensitivity.* Conclusions*. Leptin concentrations, but not HMW-adiponectin, were positively associated with foetal anthropometric measurements. Leptin concentrations are significantly associated with foetal insulin sensitivity, and there were no significant correlations between HMW-adiponectin levels and foetal insulin sensitivity.

## 1. Introduction

Currently, diabetes mellitus poses a major challenge to public health. This issue is particularly important in light of the increasing prevalence of obesity, insulin resistance, and type 2 diabetes mellitus among children and adolescents [1]. Currently, substantial epidemiological and experimental animal studies focus on the perinatal origin of vulnerability to metabolic disorders [2]. In recent years, adipokines have been introduced as novel links between obesity and its complications, including diabetes, insulin resistance, and dyslipidemia in adults and the elderly, because they are involved in the regulation of energy expenditure, appetite and satiety, insulin sensitivity, adipogenesis, fat distribution, and insulin secretion [3]. Adiponectin and leptin are two important adipokines that play a critical role in the growth and development of foetus and can regulate energy balance and insulin sensitivity in postnatal life.

Leptin is regarded as a prototypical adipokine. It can control body weight and regulate energy balance mainly through neurons in the arcuate nucleus of the hypothalamus. Furthermore, it can suppress insulin secretion from pancreatic *β* cells. Some clinical studies have shown that serum leptin concentrations are directly proportional to fat mass, and decreased central leptin responsiveness has been observed in obese patients [4]. Leptin produced by the placenta may have an important role in controlling placental growth and function. Foetal leptin is mainly derived from foetal adipose tissue. Elevated plasma leptin levels have been observed in obese and insulin resistant patients [4]. However, there is little information about the relationship between leptin and insulin resistance in neonates.

Adiponectin has been widely studied because of its diverse biological functions, including anti-inflammatory, antiatherogenic, antidiabetic, and cardioprotective effects [5]. It circulates in plasma as low-molecular-weight (LMW) trimers, medium-molecular-weight (MMW) hexamers, and high-molecular-weight (HMW) multimers. Multiple studies have indicated that HMW-adiponectin is the most active form with insulin-sensitising activity, and its circulating levels negatively correlate with obesity, insulin resistance, and coronary artery disease (CAD) [6, 7]. Substantial large-scale clinical cohorts also indicated that alterations in plasma HMW-adiponectin level may be more relevant in the prediction of insulin resistance and metabolic syndrome than total adiponectin levels [8–11]. All these evidences strongly suggest that simply measuring HMW-adiponectin would be a much more effective and sensitive biomarker than total adiponectin to evaluate the presence of insulin resistance and some metabolic disorders.

The majority of relevant studies indicated that adiponectin and leptin levels in cord blood were associated with anthropometric measurements at birth [12–14]. Moreover the negative correlation of adiponectin and HMW-adiponectin with body weight has been observed in adults and older children, whereas in neonates this correlation is positive [15]. Therefore, the relationship between plasma adiponectin concentration and anthropometric measurements is controversial. Furthermore, there is a scarcity of data on whether adipokine levels are associated with foetal insulin sensitivity. Thus, the objectives of the present study were to define the association between HMW-adiponectin, leptin, and anthropometric measurements of neonates at birth. Furthermore, we would like to explore whether HMW-adiponectin and leptin correlate with insulin sensitivity in neonates.

## 2. Subjects and Methods

### 2.1. Ethics Statement

Informed parental consent was obtained from each participant, and the study protocol was approved by the Institutional Review Board and Ethics Committee of Peking Union Medical College Hospital (PUMCH) and the Ethics Committee of PUMCH (S-002).

### 2.2. Participants

The study population consisted of 266 full-term neonates (140 males and 126 females, gestational age 37.0–41.6 weeks) consecutively born at Peking Union Medical College Hospital from July 2009 to January 2011. Infants whose mothers had multiple-fetus pregnancy, chronic hypertension, pregnancy-induced hypertension, gestational diabetes mellitus, endocrine disorders, and other severe maternal illnesses, and infants with a gestational age <37 or >42 weeks, asphyxia at birth, intrauterine infection, and congenital anomalies, were excluded from the study.

### 2.3. Anthropometric Measurement

Data and specimens were collected at delivery. Three trained research nurses and assistants collected data on maternal, pregnancy, and birth characteristics using structured study questionnaires through face-to-face interviews and medical chart reviews. The variables of the neonates included birth weight, length, head circumference, gestational age (GA), and ponderal index (PI). The clinical characteristics and anthropometric measurements are shown in Table 1. PI was calculated as body weight (kg)/body length (m^3^). The body weight of each neonate and placental weight were determined to the nearest 1 g using an electronic scale. Body length was determined to the nearest 0.1 cm in the supine position with a length board. Head circumference was determined with a plastic tape to the nearest 0.1 cm.

### 2.4. Sample Collection and Biochemical Analysis

Venous cord blood samples were obtained from 266 newborns immediately after birth with EDTA as anticoagulant. All plasmas were obtained by centrifugation at 4000 ×g for 10 min in a microcentrifuge at room temperature and stored in multiple aliquots at −80°C until biochemical assays. Plasma glucose (in mg/dL, 1 mmol/L = 18 mg/dL) was measured by an automated glucose oxidase method. Insulin was measured by a Human Insulin-Specific RIA radioimmunoassay (RIA) kit (HI-14K, Linco, St. Charles, MO). The inter- and intra-assay coefficients of variation were 2.9–6.0% and 2.2–4.4%, respectively. Leptin was measured by a human leptin RIA kit (HL-81HK, Linco, St. Charles, MO). The inter- and intra-assay coefficients of variation were 3.6–6.2% and 3.4–8.3%, respectively. The plasma HMW-adiponectin concentration was determined by a quantitative ELISA (enzyme-linked immunosorbent assay) kit (DHWAD0, R&D Systems, Minneapolis, MN). The sensitivity was 0.989 ng/mL. The intra- and interassay coefficients of variation were 2.6–3.7% and 8.3–8.6%, respectively. All samples were tested in duplicate in a blinded manner. Although the euglycaemic insulin clamp technique is the best available standard for measurement of insulin action, it was impossible to apply at birth. The cord plasma G/I (glucose/insulin ratio), which is a simple estimate of insulin sensitivity using insulin and glucose levels [16, 17], was used as a surrogate indicator of foetal insulin sensitivity. G/I was calculated as G (mg/dL)/I (*μ*U/mL).

### 2.5. Statistical Analysis

The data were expressed as the mean ± standard deviation (SD). We compared baseline characteristics between male and female groups using an unpaired *t*-test or the Mann-Whitney *U* test. Distributions were tested for normality using the Shapiro-Wilk *W* test. Log transformations were applied for variables with skewed data distribution in all comparisons before correlation and regression analyses. Pearson correlation analysis was used to assess the relationships between HMW-adiponectin and some anthropometric measurements and biochemical characteristics. Multiple regression analysis assessed whether the relationship between leptin and insulin sensitivity is independent of the other clinical parameters. Two-tailed *P* values <0.05 were considered statistically significant. All statistical analyses were calculated with SPSS 15.0 (SPSS, Inc., Chicago, IL, USA).

## 3. Results

### 3.1. Biochemical Characteristics and Sexual Dimorphism

HMW-adiponectin is present abundantly in cord blood. Cord blood concentrations of HMW-adiponectin, glucose, insulin, and G/I are shown in Table 2. Regarding anthropometric measurements, birth weight (3436.8 ± 483.96 versus 3303.21 ± 442.7, *P* = 0.02), body length (49.86 ± 1.87 versus 49.26 ± 1.95, *P* = 0.01), and birth weight to length ratio (68.72 ± 7.80 versus 66.54 ± 9.72, *P* = 0.04) were significantly higher in male than female subjects. However, females had higher HMW-adiponectin and leptin concentrations compared with males (15.35 ± 6.67 versus 13.34 ± 6.77, *P* = 0.031, and 10.82 ± 6.32 versus 8.12 ± 5.12, *P* = 0.000, resp.). No significant differences were detected in insulin sensitivity between male and female neonates.

### 3.2. Univariate Correlations

Univariate correlation analysis showed that leptin concentrations in cord blood were positively associated with gestational age (*r* = 0.24, *P* = 0.001), birth weight (*r* = 0.391, *P* = 0.000), body length (*r* = 0.28, *P* = 0.000), ponderal index (*r* = 0.242, *P* = 0.000), and placenta weight (*r* = 0.323, *P* = 0.000). Furthermore, leptin concentrations were positively associated with insulin (*r* = 0.407, *P* = 0.000) and negatively associated with G/I (*r* = −0.304, *P* = 0.000). However, there was no correlation between cord blood HMW-adiponectin levels and foetal anthropometric measurements (all *P* > 0.05). HMW-adiponectin concentrations in cord blood were also positively associated with insulin (*r* = 0.223, *P* = 0.001) and leptin (*r* = 0.151, *P* = 0.028). However, no correlation was found between cord blood HMW-adiponectin levels and foetal insulin sensitivity indicator (*P* > 0.05) (Table 3).

### 3.3. Multivariate Linear Regression Analysis

Having demonstrated that leptin in cord blood is positively associated with insulin sensitivity in the foetus at birth, we next queried whether the relationship between leptin and insulin sensitivity is independent of other clinical parameters. Multivariate stepwise regression analysis with logarithmically transferred G/I as a dependent variable was applied to investigate the relationship. Leptin (*B* = −0.126, *P* = 0.045) was independently associated with insulin sensitivity as estimated by G/I after adjusting for HMW-adiponectin, gestational age, birth weight, body length, head circumference, placenta weight, and the type of delivery (Table 4).

## 4. Discussion

To our knowledge, so far, our present study has relatively more clinical foetal samples compared with any other study, which could indicate a more convincing result [13, 18–20]. Here we show that HMW-adiponectin is present abundantly in cord blood, and cord blood HMW-adiponectin levels were significantly higher compared with adult levels, consistent with other studies [9, 19]. The high foetal HMW-adiponectin levels may be largely attributable to synthesis by adipocytes. HMW-adiponectin is also produced by the placenta and various foetal tissues, such as, skeletal muscle, skin, small intestine, amniotic membrane, and placenta. This is in contrast to its secretion by adipose tissue exclusively in adult humans [21, 22].

In our present study, the results indicated that HMW-adiponectin was significantly higher in females compared with males. Similar results were shown in another study [23]. A recent study focused on the gender dimorphism of adiponectin multimers demonstrated that the cord blood LMW isoform emerged as the main determinant of subcutaneous fat mass in male neonates, whereas cord blood insulin and the HMW isoform were more relevant in female neonates [24]. The differences in cord leptin concentrations between male and female neonates may be explained by differences in fat mass [25] and sex hormone levels, such as testosterone [26]. We also found that cord plasma leptin concentrations were significantly higher in female newborns than those in male newborns, which is consistent with the results of previous studies [12, 13, 25]. In our present study, there was no correlation between cord blood HMW-adiponectin levels and foetal anthropometric measurements. Similarly, such relationships were also not found in other studies [22, 27]. It has been previously shown that being born small for gestational age (SGA) is an independent negative predictor of circulating adiponectin levels [28]. The inclusion of SGA infants in our study might be an explanation for the absence of the expected positive correlation between adiponectin levels and anthropometric measurements. Conversely, some studies indicated that adiponectin and leptin levels in cord blood were associated with anthropometric measurements at birth [12–14]. This relationship may be affected by the clinical samples and the healthy status of the pregnant women.

The results of our present study also indicated that leptin levels in cord blood are negatively correlated with foetal insulin sensitivity. Consistently, two other studies focused on the foetal leptin and insulin sensitivity, which also demonstrated a negative correlation [29, 30]. The negative association may be partly explained by the amount of adipose tissue in the foetus. Therefore, our finding suggests that leptin may play an important role in metabolic homeostasis during foetal life. Our study detected the relationship between HMW-adiponectin levels in cord blood and foetal insulin sensitivity at birth. Unexpectedly, no correlation was found between cord blood HMW-adiponectin levels and foetal insulin sensitivity indicator. As described previously, two studies also indicated that there was no significant correlation between cord blood adiponectin and foetal insulin sensitivity [18, 30]. Although HMW-adiponectin is a better indication of biologically active adiponectin, it cannot adequately reflect the effects of metabolic events in the newborn. There may be several explanations for this. First, because of significantly higher HMW-adiponectin concentrations in cord blood, which has been reported to be fivefold higher in cord blood than in adult blood, it may have reached a relatively high “saturation” level in foetal circulation. Furthermore, insulin resistance may be several orders of magnitude lower in newborns than in adults, which suggests a state of higher insulin sensitivity in utero [20]. An alternative explanation is that as it is a cross-sectional study it cannot reflect a causal relationship between HMW-adiponectin levels in cord blood and foetal insulin sensitivity. Therefore, the long-term implications of foetal HMW-adiponectin level for future metabolic health remain to be understood.

## 5. Conclusions

Our findings suggest that leptin, but not HMW-adiponectin, is a useful biomarker for the evaluation of the metabolic status of neonates, distinct from that of adults. Adipokines may play a critical role in the growth, development, and glucose metabolism of the neonates.

## Figures and Tables

**Table 1:** Clinical characteristics and anthropometric measurements of newborns.

	Males (*n* = 140)	Females (*n* = 126)	All subjects (*n* = 266)	*P* value
Gestational age (weeks)	39.01 ± 0.89	39.22 ± 0.89	39.11 ± 0.90	0.127
Birth weight (g)	3436.8 ± 483.96	3303.21 ± 442.7	3373.53 ± 468.79	0.02*
Body length (cm)	49.86 ± 1.87	49.26 ± 1.95	49.58 ± 1.93	0.01*
Ponderal index (kg/m^3^)	27.59 ± 2.25	27.67 ± 2.94	27.62 ± 2.59	0.812
Birth weight to length ratio (g/cm)	68.72 ± 7.80	66.54 ± 9.72	67.69 ± 8.81	0.04*
Head circumference (cm)	34.29 ± 1.45	34.00 ± 1.12	34.16 ± 1.30	0.07
Placenta weight (g)	670.36 ± 141.19	652.04 ± 125.49	661.58 ± 133.93	0.291

Data reported as the mean ± SD. *P < 0.05 comparing female versus male newborns as assessed by Mann-Whitney *U* test.

**Table 2:** Biochemical characteristics and estimates of insulin sensitivity in cord blood at birth.

	Males (*n* = 140)	Females (*n* = 126)	All subjects (*n* = 266)	*P* value
Blood glucose (mg/dL)	76.16 ± 22.07	81.50 ± 18.28	78.68 ± 20.47	0.095
Insulin (*µ*U/mL)	13.08 ± 5.99	13.63 ± 5.94	13.34 ± 5.96	0.458
Leptin (ng/mL)	8.12 ± 5.12	10.82 ± 6.32	9.40 ± 5.87	0.000*
HMW-adiponectin (*µ*g/mL)	13.34 ± 6.77	15.35 ± 6.67	14.82 ± 7.24	0.031*
Insulin sensitivity index				
G/I	7.29 ± 4.61	6.88 ± 3.99	7.11 ± 4.32	0.551

Data reported as the mean ± SD. *P < 0.05 versus males as assessed by Mann-Whitney *U* test. G/I: glucose/insulin ratio.

**Table 3:** Univariate correlation coefficients.

	HMW-adiponectin		Leptin	
	*r*	*P*	*r*	*P*
Gestational age	−0.014	0.844	0.24	0.001**
Birth weight	−0.052	0.449	0.391	0.000***
Body length	−0.114	0.10	0.28	0.000***
Ponderal index	0.036	0.606	0.242	0.000**
Placenta weight	−0.130	0.072	0.323	0.000***
Insulin	0.223	0.001**	0.407	0.000**
Leptin	0.151	0.028*	—	—
HMW-adiponectin	—	—	0.151	0.028*
Insulin sensitivity index				
G/I	0.225	0.215	−0.304	0.000***

*P < 0.05, **P < 0.01, and ***P < 0.001. G/I: glucose/insulin ratio.

**Table 4:** Multivariate stepwise linear regression analysis with insulin sensitivity index (G/I) as dependent variable.

Independent variable	Beta coefficient	Standard error	*t*	*P* value
Leptin	−0.126	0.063	−1.502	0.045*
Birth weight	−0.004	0.001	−5.057	0.000***
HMW-adiponectin	−0.167	0.053	−3.152	0.08

Model adjusted *R* ^2^ = 0.251. Independent variables included in the model were HMW-adiponectin (*µ*g/mL), leptin (ng/mL), gestational age (weeks), birth weight (g), body length (cm), head circumference (cm), placenta weight (g), and the type of delivery. *P < 0.05, ***P < 0.001. G/I: glucose/insulin ratio; HMW-adiponectin: high-molecular-weight adiponectin.

## References

[1] Pinhas-Hamiel O, Zeitler P (2005). The global spread of type 2 diabetes mellitus in children and adolescents. *Journal of Pediatrics*.

[2] Chavatte-Palmer P, Tarrade A, Lévy R (2012). Developmental origins of health and disease in adults: role of maternal environment. *Gynecologie Obstetrique Fertilite*.

[3] Scherer PE (2006). Adipose tissue: from lipid storage compartment to endocrine organ. *Diabetes*.

[4] Zhou Y, Rui L (2013). Leptin signaling and leptin resistance. *Frontiers of Medicine*.

[5] Mattu HS, Randeva HS (2013). Role of adipokines in cardiovascular disease. *Journal of Endocrinology*.

[6] Hara K, Horikoshi M, Yamauchi T (2006). Measurement of the high-molecular weight form of adiponectin in plasma is useful for the prediction of insulin resistance and metabolic syndrome. *Diabetes Care*.

[7] Eglit T, Lember M, Ringmets I, Rajasalu T (2013). Gender differences in serum high-molecular-weight adiponectin levels in metabolic syndrome. *European Journal of Endocrinology*.

[8] Hirose H, Yamamoto Y, Seino-Yoshihara Y, Kawabe H, Saito I (2010). Serum high-molecular-weight adiponectin as a marker for the evaluation and care of subjects with metabolic syndrome and related disorders. *Journal of Atherosclerosis and Thrombosis*.

[9] Kizer JR, Barzilay JI, Arnold AM (2012). Total and high-molecular-weight adiponectin and risk of incident diabetes in older people. *Diabetes Care*.

[10] Sulistyoningrum DC, Gasevic D, Lear SA, Ho J, Mente A, Devlin AM (2013). Total and high molecular weight adiponectin and ethnic-specific differences in adiposity and insulin resistance: a cross-sectional study. *Cardiovascular Diabetology*.

[11] Seino Y, Hirose H, Saito I, Itoh H (2007). High molecular weight multimer form of adiponectin as a useful marker to evaluate insulin resistance and metabolic syndrome in Japanese men. *Metabolism: Clinical and Experimental*.

[12] Sivan E, Mazaki-Tovi S, Pariente C (2003). Adiponectin in human cord blood: relation to fetal birth weight and gender. *The Journal of Clinical Endocrinology & Metabolism*.

[13] Inoue M, Itabashi K, Nakano Y, Nakano Y, Tobe T (2008). High-molecular-weight adiponectin and leptin levels in cord blood are associated with anthropometric measurements at birth. *Hormone Research*.

[14] Kotani Y, Yokota I, Kitamura S, Matsuda J, Naito E, Kuroda Y (2004). Plasma adiponectin levels in newborns are higher than those in adults and positively correlated with birth weight. *Clinical Endocrinology*.

[15] Siahanidou T, Margeli A, Garatzioti M (2009). Disparity in circulating adiponectin multimers between term and preterm infants. *Journal of Perinatal Medicine*.

[16] Gungor N, Saad R, Janosky J, Arslanian S (2004). Validation of surrogate estimates of insulin sensitivity and insulin secretion in children and adolescents. *Journal of Pediatrics*.

[17] Luo Z-C, Delvin E, Fraser WD (2010). Maternal glucose tolerance in pregnancy affects fetal insulin sensitivity. *Diabetes Care*.

[18] Basu S, Laffineuse L, Presley L, Minium J, Catalano PM, Hauguel-de Mouzon S (2009). In utero gender dimorphism of adiponectin reflects insulin sensitivity and adiposity of the fetus. *Obesity*.

[19] Odden N, Mørkrid L (2007). High molecular weight adiponectin dominates in cord blood of newborns but is unaffected by pre-eclamptic pregnancies. *Clinical Endocrinology*.

[20] Pinar H, Basu S, Hotmire K (2008). High molecular mass multimer complexes and vascular expression contribute to high adiponectin in the fetus. *Journal of Clinical Endocrinology and Metabolism*.

[21] Chen J, Tan B, Karteris E (2006). Secretion of adiponectin by human placenta: differential modulation of adiponectin and its receptors by cytokines. *Diabetologia*.

[22] Corbetta S, Bulfamante G, Cortelazzi D (2005). Adiponectin expression in human fetal tissues during mid- and late gestation. *Journal of Clinical Endocrinology and Metabolism*.

[23] Lau SM, Hng TM, de Vries B, McLean M, Cheung NW (2009). Sexual dimorphism of high molecular weight adiponectin in cord blood. *Clinical Endocrinology*.

[24] Simón-Muela I, Näf S, Ballesteros M (2013). Gender determines the actions of adiponectin multimers on fetal growth and adiposity. *The American Journal of Obstetrics and Gynecology*.

[25] Petridou E, Mantzoros CS, Belechri M (2005). Neonatal leptin levels are strongly associated with female gender, birth length, IGF-I levels and formula feeding. *Clinical Endocrinology*.

[26] Ertl T, Funke S, Sárkény I (1999). Postnatal changes of leptin levels in full-term and preterm neonates: their relation to intrauterine growth, gender and testosterone. *Biology of the Neonate*.

[27] Lindsay RS, Walker JD, Havel PJ, Hamilton BA, Calder AA, Johnstone FD (2003). Adinopnectin is present in cord blood but is unrelated to birth weight. *Diabetes Care*.

[28] Siahanidou T, Mandyla H, Papassotiriou G-P, Papassotiriou I, Chrousos G (2007). Circulating levels of adiponectin in preterm infants. *Archives of Disease in Childhood: Fetal and Neonatal Edition*.

[29] Catalano PM, Presley L, Minium J, Mouzon SHD (2009). Fetuses of obese mothers develop insulin resistance in utero. *Diabetes Care*.

[30] Luo Z-C, Nuyt A-M, Delvin E (2013). Maternal and fetal leptin, adiponectin levels and associations with fetal insulin sensitivity. *Obesity*.

